# Understanding Anastomotic Healing in Colo-Rectal Surgery; a Multicentric 5-Year Analysis of Predictive Factors for Integrity and Fistula Formation

**DOI:** 10.3390/diagnostics16060837

**Published:** 2026-03-11

**Authors:** Dumitru-Dragos Chitca, Octavian Mihalache, Florin Bobircă, Cristian Botezatu, Valentin Popescu, Dan Andras, Maria-Theodora Lapadat, Martina Nichilo, Dragos Eugen Georgescu, Petronel Mustățea, Horia Doran, Bogdan Mastalier, Traian Pătrașcu

**Affiliations:** 1 General Surgery Department, Carol Davila University of Medicine and Pharmacy, 050474 Bucharest, Romania; dumitru-dragos.chitca@umfcd.ro (D.-D.C.); octavian.mihalache@umfcd.ro (O.M.); popescu.vali.umf@gmail.com (V.P.); danandras69@gmail.com (D.A.); martina.nichilo@rez.umfcd.ro (M.N.); dragos-eugen.georgescu@umfcd.ro (D.E.G.); petronel.mustatea@umfcd.ro (P.M.); horia.doran@umfcd.ro (H.D.); bogdanmastalier@yahoo.com (B.M.); traian.patrascu@umfcd.ro (T.P.); 2 General Surgery Clinic, “Colentina” Clinical Hospital, 020125 Bucharest, Romania; teodoralpd@gmail.com; 3 Surgical Department I, “Dr. I. Cantacuzino” Clinical Hospital, 030167 Bucharest, Romania; 4 Surgery Department, Municipal Hospital Urziceni, 925300 Urziceni, Romania

**Keywords:** colorectal surgery, anastomotic leak, ASA score, risk factors, surgical outcomes

## Abstract

**Background**: Anastomotic leakage (AL) remains one of the most feared complications after colorectal surgery. This study aimed to identify preoperative risk factors for AL using a five-year dataset from two Romanian surgical clinics. **Materials and Methods**: A retrospective cohort of 155 patients undergoing colorectal resection with primary anastomosis (105 from “Colentina” Hospital and 50 from “Dr. I. Cantacuzino” Hospital) was analyzed. Preoperative demographic, clinical, and laboratory data were extracted and assessed using univariate and multivariable logistic regression. Statistical analyses were performed using IBM SPSS. **Results**: The overall AL rate was 10.3%. Multivariable analysis identified high ASA class (OR 17.6; *p* = 0.001), emergency surgery (OR 32.2; *p* = 0.0007), and heavy alcohol use (OR 15.3; *p* = 0.004) as independent predictors of leakage. While low preoperative albumin and smoking were associated with leakage in a bivariate analysis, these did not remain significant after adjustment. Notably, all laboratory markers were based on preoperative values, distinguishing our approach from prior studies that commonly evaluated postoperative biomarkers. No statistically significant effect was found for neoadjuvant chemotherapy or radiotherapy after controlling for other covariates. **Conclusions**: High ASA score, alcohol abuse, and emergency surgery were the strongest independent predictors of AL in our cohort. The lack of predictive power of certain widely reported factors, such as low albumin, may reflect our dataset’s focus on preoperative optimization. These findings support the use of individualized risk assessment and reinforce the role of preoperative preparation in reducing leak incidence in colorectal surgery.

## 1. Introduction

Anastomotic leakage (AL) is a significant complication following colorectal resection, with reported occurrences typically ranging from 2% to 19%, while some studies indicate a variation from 6% to 30%, with a larger prevalence in low rectal anastomoses compared to colonic resections [1,2,3,4,5,6,7,8,9,10]. It is persistently linked to heightened morbidity, mortality, and reoperation rates; permanent stoma formation; and poorer oncological outcomes in rectal cancer [11,12].

Recent narrative and systematic reviews categorize risk factors into non-modifiable, modifiable, and technical/surgical categories. Non-modifiable characteristics encompass male sex, substantial comorbidity burden (cardiovascular, pulmonary, diabetes), and advanced tumor stage; a low rectal tumor placement and minimal distance from the anal margin are consistently associated with elevated leak rates [13,14,15].

Surgical factors, particularly anastomotic height, are crucial determinants; leak rates can reach approximately 19% for low rectal (coloanal) anastomoses, in contrast to about 1–3% for more proximal colon anastomoses [16,17]. The choice of surgical method (laparoscopic versus open) has negligible effect on the risk of leaking, as studies indicate comparable incidence rates of leaks across minimally invasive and open resections [18]. Utilization of a protective diverting stoma significantly diminishes anastomotic leakage in low anterior resection, according to research [19]. A meta-analysis indicates that clinically significant leaks occur in approximately 6% of diverted patients, compared to around 18% in those without diversion (*p* < 0.00001) [20]. Transanal decompression tubes (TDTs) have been investigated as a beneficial, safe, prophylactic intervention, yielding conflicting outcomes. TDTs are intended to facilitate endo-luminal pressure reduction and fecal diversion, hence providing a protective effect on anastomotic healing. Some analyses indicate a reduction in leak rates, whilst others demonstrate no substantial overall advantage to the routine application of TDTs [21,22,23,24,25,26,27,28,29,30,31,32].

Equally crucial is the establishment of a tension-free, well-perfused anastomosis, as insufficient perfusion or high tension at the site significantly heightens the risk of leakage [33].

Intraoperative perfusion evaluation, such as that using indocyanine green fluorescence, correlates with markedly reduced leak rates by verifying sufficient blood flow prior to finalizing the anastomosis [33,34,35,36,37].

The anastomotic technique (hand-sewn versus stapled) does not significantly affect leak incidence; recent extensive research and reviews have demonstrated no difference in leak rates between stapled and hand-sutured anastomoses [38,39,40,41].

Compliance with ERAS guidelines and the volume of procedures performed by the hospital or surgeon also affect risk, indicating that systemic factors and standardized pathways can reduce patient- and tumor-related risks [15,42,43].

Alterable patient-related factors encompass smoking, excessive alcohol intake, obesity, malnutrition, and immunosuppression (due to steroids and certain biologics), in addition to exposure to preoperative radiation and perioperative blood transfusions [13,44,45,46].

Extensive cohort studies and meta-analyses validate that male gender, obesity/high BMI, diabetes, pulmonary disease, and elevated ASA class are independent predictors of anastomotic leakage following colon or colorectal cancer surgery [14,42,46].

Reduced blood albumin levels and several indicators of malnutrition are increasingly recognized as significant predictors of leakage in rectal cancer, emphasizing the necessity of prior rehabilitation and nutritional enhancement [45,47].

Despite extensive research on anastomotic leakage, important gaps persist. Many predictive models include heterogeneous surgical populations and rely predominantly on postoperative biomarkers—particularly dynamic CRP and albumin changes—thereby identifying leakage after inflammatory processes have already begun. In contrast, the independent prognostic value of strictly preoperative laboratory parameters, measured prior to surgical stress and systemic inflammatory activation, remains insufficiently defined. Furthermore, existing analyses often derive from single-center reports or large registries without accounting for structured preoperative optimization in tertiary care settings.

This multicentric five-year study addresses this gap by exclusively evaluating preoperative demographic, clinical, and laboratory variables in two clinical hospitals. By isolating baseline physiological status from postoperative biomarker kinetics, we aim to refine preoperative risk stratification and determine whether traditional laboratory predictors retain independent prognostic relevance when assessed before surgical intervention.

## 2. Materials and Methods

This study was designed as a retrospective, multicenter analysis conducted at two surgical centers in Bucharest, Romania. These included all the surgical patients of “Colentina” Clinical Hospital and the patients from General Surgery Clinical Ward I of “Dr. I. Cantacuzino” Clinical Hospital. All adult patients (aged 18 years and older) who underwent an anterior colorectal resection with a primary colorectal anastomosis between January 2020 and December 2024 were identified through hospital databases, operative logs and patients’ charts. This time frame was chosen to provide a 5-year experience from both centers. The study focused on colorectal anastomotic healing and fistula formation (anastomotic leakage, AL).

The exclusion criteria included surgeries without a primary anastomosis (e.g., those ending in an end stoma); procedures performed solely for palliation (e.g., diversion for advanced disease without an anastomosis); and cases with incomplete perioperative records. These criteria ensured that only cases with complete anastomotic data were analyzed. After these criteria were applied, all remaining eligible patients from both institutions were included in the study cohort.

Clinical data were extracted retrospectively from patient medical charts, operative reports, and laboratory records at each hospital. Data collection was standardized between the two centers to ensure consistency. The preoperative variables obtained included patient demographics and health status: age, sex, body mass index (BMI), major comorbidities (such as diabetes and cardiovascular disease), preoperative ASA (American Society of Anesthesiologists) physical status score, smoking status, and alcohol use. In addition, key preoperative laboratory values were recorded for each patient, all of which were measured within 48 h prior to surgery. These lab tests included those for hemoglobin levels, white blood cell (WBC) counts, serum creatinine, coagulation parameters (including international normalized ratio, INR), C-reactive protein (CRP), serum albumin, and total protein levels.

Intraoperative variables were documented based on operative reports. These included the type of resection performed and the anatomical level of the anastomosis (categorized as high, middle, or low rectal anastomosis based on the distance from the anal verge). Surgical urgency was noted as elective or emergency surgery. The surgical approach was recorded as open vs. laparoscopic (both centers do not have access to robotic-assisted surgery). The anastomosis technique was classified as hand-sewn (manual suturing) versus stapled anastomosis. We also recorded whether the patient received perioperative blood transfusions and whether a protective stoma was created during the initial surgery. Additionally, at the “Dr. I. Cantacuzino” center, it was routine practice to place a transanal decompression tube for low anastomoses; the use of a transanal drainage tube was therefore captured as a variable (yes—1/no—0) for each case.

For each patient, the primary outcome of interest was the occurrence of an AL (anastomotic dehiscence/fistula formation) in the postoperative period. AL was defined as any clinical or radiologic evidence of an anastomotic dehiscence leading to intra-abdominal infection, fecal discharge from drains, or a need for re-intervention. The presence or absence of AL was determined from postoperative clinical notes, imaging reports, and intervention records. For cases of leakage, we also noted the timing of leak detection (categorized as early, within 7 days postoperatively, or late, after 7 days) as documented in the charts.

All data were entered into a secure database and analyzed using IBM SPSS v26.0 Statistics software. Descriptive statistics were used to summarize the patient cohort and operative details. Categorical variables were expressed as frequencies or percentages, while continuous variables were summarized by their mean and standard deviation or median and interquartile range, as appropriate. Office Excel was used for visual representation of the statistical analysis.

Cases with incomplete perioperative records were excluded during cohort selection. After database cleaning, no variable-level missing data remained for the variables included in the final regression analyses; therefore, a complete-case analysis approach was applied without imputation.

The variables considered for multivariable logistic regression were selected based on a combination of clinical relevance and statistical significance in a univariate analysis. Factors demonstrating a *p*-value < 0.10 in univariate testing were considered for model inclusion, together with variables consistently reported in the literature as established risk factors for anastomotic leakage (e.g., ASA class, emergency surgery, and neoadjuvant therapy), irrespective of their univariate *p*-value.

To avoid overfitting given the limited number of leak events, the final multivariable model was constructed using a parsimonious approach, retaining only clinically meaningful variables while ensuring an appropriate events-per-variable ratio. Adjusted odds ratios (ORs) with 95% confidence intervals (CIs) are reported.

The ASA physical status classification was treated as a categorical variable for descriptive analyses. For regression modeling, ASA classes were dichotomized into low-risk (ASA I–II) versus high-risk (ASA III–V) categories to enhance clinical interpretability and to preserve statistical stability given the limited number of outcome events.

## 3. Results

**Figure 1:**
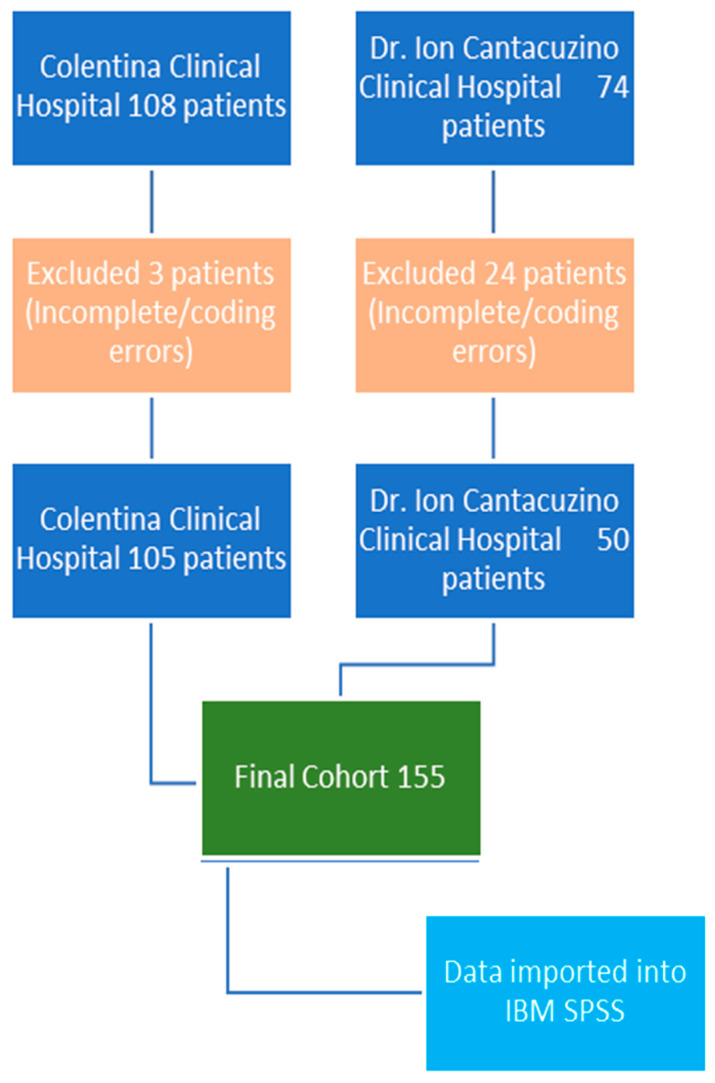
Patient inclusion process.

After the initial hospital database patient search, we identified 108 potential patients from “Colentina” Clinical Hospital and 74 potential patients from “Dr. I. Cantacuzino” Clinical Hospital. After data collection, charts and operation logs review, three patients from “Colentina” were excluded for incomplete records and coding error and 24 patients were excluded from the “Dr. I. Cantacuzino” database for coding inconsistencies and incomplete data (Figure 1).

**Figure 2:**
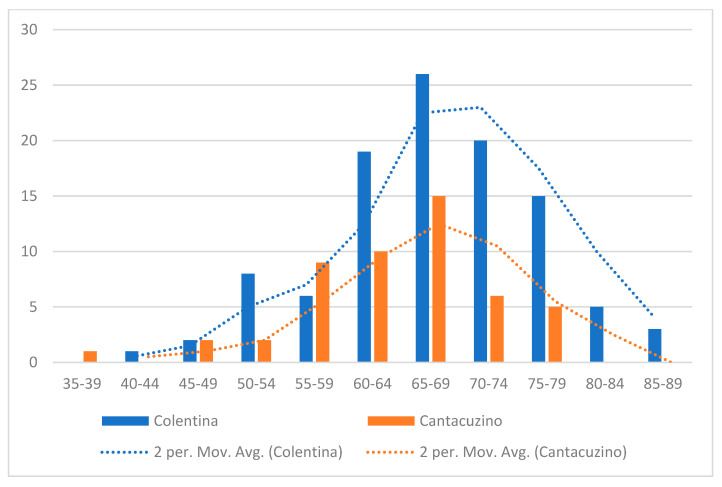
Distribution of age by center.

“Colentina” patients were slightly older (mean 67.4 ± 9.0 vs. 64.0 ± 8.2 years, *p* ≈ 0.021) (Figure 2) and had more laparoscopic procedures (38.5% vs. 6.1% laparoscopic, *p* < 0.001). “Dr. I. Cantacuzino” patients more often received neoadjuvant therapy (chemotherapy: 33% vs. 15%, *p* ≈ 0.025; radiotherapy: 40.8% vs. 15.4%, *p* ≈ 0.001).

The sex distribution and rates of comorbidities were similar across both centers.

**Figure 3:**
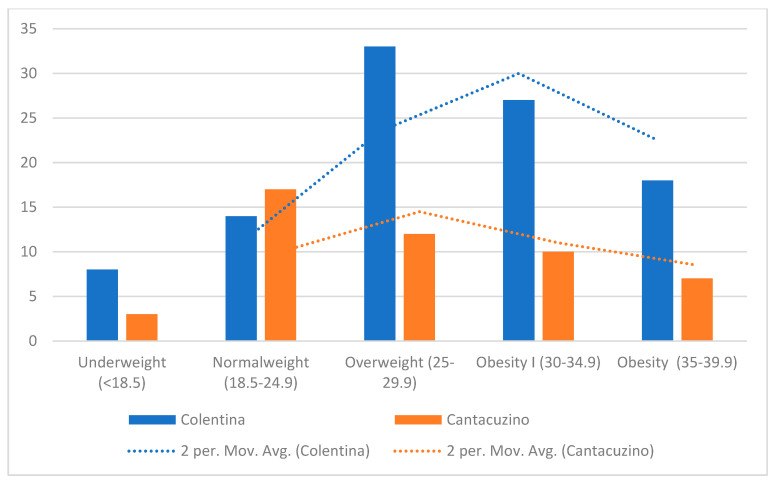
Distribution of BMI by center.

The “Colentina” center demonstrated a higher concentration of patients with BMIs above the population median (especially in the 30–35 kg/m^2^ range), reflecting a higher prevalence of overweight and potentially obese patients (Figure 3). By contrast, the “Dr. I. Cantacuzino” BMI distribution shows wider variance and a heavier tail toward lower BMI categories (<25 kg/m^2^), including several outliers below 20 kg/m^2^. These findings suggest demographic or referral pattern differences between the centers.

While both distributions peak around 27–30 kg/m^2^, the Gaussian kernel density estimate (KDE) for “Colentina” is unimodal and sharper, indicating tighter clustering around the mean, whereas the “Dr. I. Cantacuzino” KDE is flatter, suggesting more heterogeneity in body habitus.

Other preoperative factors (anemia, leukocytosis, platelet count, CRP, albumin, protein, creatinine, INR abnormalities) were similar in both patient cohorts.

The overall AL rates were comparable (Colentina 10.6%, Cantacuzino 10.2%, *p* ≈ 1.00).

As shown in Table 1, baseline demographic characteristics and major comorbidities were comparable between the centers. The only significant inter-institutional differences were observed in age, neoadjuvant therapy rates, and surgical approach, while overall leak incidence did not differ.

**Figure 4:**
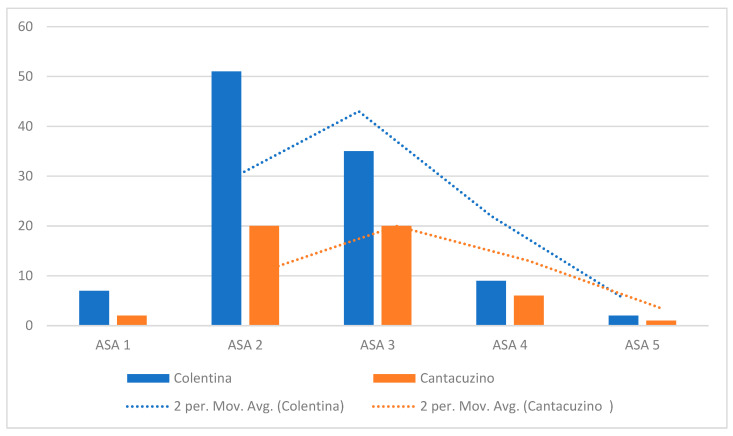
Distribution of ASA classes by center.

The “Colentina” center’s case mix is skewed toward lower-risk patients, with the majority of cases classified as ASA I–II (Figure 4). Specifically, ASA I and II collectively account for nearly 70% of the “Colentina” cohort. In contrast, “Dr. I. Cantacuzino” presents a more even distribution across ASA classes I–III, with notably higher proportions of ASA III patients and a small but visible representation of ASA IV. ASA V appears rarely in either group but is not absent.

The kernel density estimate (KDE) curves further emphasize the divergence in patient complexity: the “Colentina” curve is left-shifted and peaks at ASA II, while that for “Dr. I. Cantacuzino” is flatter, with a greater density at ASA III and a longer tail extending toward higher-risk strata.

This finding is consistent with previous intercenter comparisons and reflects a differing case profile between the two institutions. The higher rates of open surgical approaches and neoadjuvant treatments observed at “Dr. I. Cantacuzino” may be influenced by a greater proportion of lower rectal tumors and tailored treatment strategies, rather than necessarily indicating a higher overall case complexity.

Across both centers, the overall AL rate was 10.3% (16/155). Of these, 12 cases (75%) were identified as early leaks (within 7 days postoperatively), while four cases (25%) occurred later.

We analyzed the datasets to assess each blood marker’s association with leak risk. For each variable (hemoglobin, WBCs, CRP, albumin, total protein, creatinine, INR), we fit a univariate logistic regression (outcome = leak yes/no) to estimate the odds ratio (OR) and *p*-value (Table 2).

Of the eight markers, none showed a statistically significant association in our data, but the point estimates suggest possible trends. Hemoglobin had OR ≈ 1.58 (95% CI 0.41–6.12, *p* = 0.51), indicating a non-significant trend toward higher leak risk with higher hemoglobin. WBC count had OR ≈ 1.68 (95% CI 0.43–6.54, *p* = 0.45), which was also non-significant but elevated preoperatively (Table 2).

CRP had an estimated OR ≈ 1.17 (95% CI 0.14–10.0, *p* = 0.89) for each unit increase, i.e., a negligible effect. In our data, preoperative CRP did not significantly predict leakage.

Albumin showed OR ≈ 2.41 (95% CI 0.25–23.1, *p* = 0.45), although this effect was not significant on AL prediction.

Our data trend hints that poorer nutritional status (lower albumin or total protein) may impair anastomotic healing. Total protein had OR ≈ 1.03 (95% CI 0.12–8.75, *p* = 0.98), showing no effect.

Creatinine gave OR ≈ 1.66 (95% CI 0.49–5.64, *p* = 0.42) per unit; elevated creatinine may reflect comorbidity or poor perfusion, but no association was found here.

The INR had OR ≈ 4.89 (95% CI 0.42–57.4, *p* = 0.21) per unit increase, with very wide confidence limits. A higher INR (coagulopathy) might theoretically impair healing or signal liver disease, but our CI spanned unity due to the limited sample size, so this was not statistically significant.

We found no blood marker with a significant predictive effect on leak risk, likely reflecting the small event numbers.

These findings suggest that in our cohort of elective colorectal cases, no single preoperative blood parameter was a significant univariate predictor of anastomotic leak. For clinical interpretation, markers of inflammation (WBCs, CRP) and nutrition (albumin, total protein) remain important.

In univariate logistic regression analysis, emergency surgery emerged as a strong and statistically significant predictor of anastomotic leakage, with an estimated odds ratio (OR) of approximately 17.0 (95% CI: 2.58–111.9, *p* ≈ 0.003), indicating a markedly elevated risk compared to elective procedures. Neoadjuvant radiotherapy was also significantly associated with increased leakage risk (OR ≈ 3.29, 95% CI: 1.10–9.82, *p* ≈ 0.033), while neoadjuvant chemotherapy showed a positive trend toward higher leakage risk but did not reach statistical significance (OR ≈ 2.87, 95% CI: 0.94–8.78, *p* ≈ 0.064). Other examined variables—including sex, comorbidity burden, and preoperative anemia—did not demonstrate significant associations with leakage (all *p* > 0.1).

**Figure 5:**
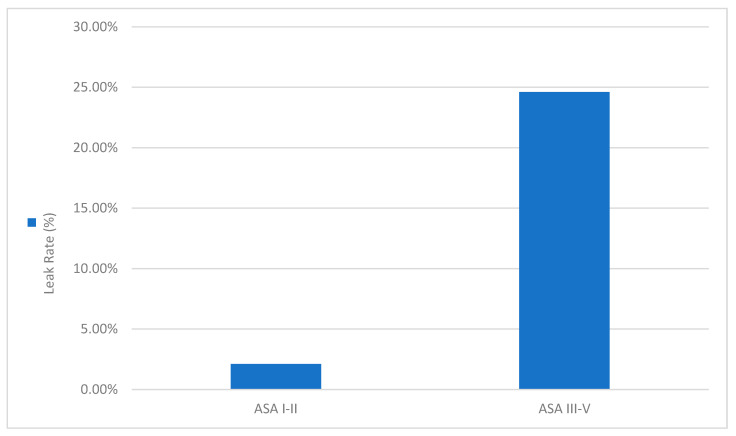
AL rate by ASA physical status.

Figure 5 depicts the sharp contrast in leak incidence by ASA class, reaffirming previous findings that high ASA status correlates with poor healing capacity. Higher ASA status (III–V) was associated with significantly greater AL incidence (~24.6%) as compared to ASA I–II (~2.1%).

In multivariable logistic regression (Table 3), emergency surgery remained the only independent predictor of leakage. Neoadjuvant radiotherapy and chemotherapy lost statistical significance after adjustment.

When outcomes were compared by institution, no significant difference in AL rates was found: “Colentina” (10.6%) vs. “Dr. I. Cantacuzino” (10.2%), *p* = 1.00. This suggests that, despite differences in surgical approach and protective strategies, overall anastomotic integrity was comparable between the centers.

## 4. Discussion

Recent thorough studies and evaluations indicate that anastomotic leak rates in colorectal surgery typically vary from approximately 2% to 19% overall [1,2,3,4,5,6,7,8,9,10]. This broad spectrum indicates variations in patient demographics, surgical circumstances (elective versus emergency), and particularly the site of anastomosis [48,49,50,51]. Our data fall in this wide range offered by previous studies regarding overall AL rates.

Preoperative anemia has been recognized as an independent risk factor for colorectal anastomotic leak (AL). Patients with low preoperative hemoglobin exhibit significantly elevated rates of anastomotic leakage, with one study indicating around a 5-fold increase in odds of leakage and another finding a roughly 6.5-fold increased risk when hemoglobin levels are below 11 g/dL [52,53,54].

Our study examined the prognostic capacity of preoperative blood tests regarding AL rates. Hemoglobin exhibited an odds ratio (OR) of around 1.58 (95% confidence interval [CI] 0.41–6.12, *p* = 0.51), while white blood cell (WBC) count showed an OR of approximately 1.68 (95% CI 0.43–6.54, *p* = 0.45), both findings being non-significant, although increased preoperative WBC count has been identified as an independent risk factor in other studies [55].

In our study, CRP had a minimal impact on AL. CRP is an acute-phase inflammatory marker frequently enhanced in the context of a potential leak; for instance, significantly higher postoperative CRP levels (>180 mg/L on day 4) are recognized as predictors of leaks [56]. In our data, preoperative CRP did not significantly predict leak, possibly because postop trends are more informative.

Total protein had OR ≈ 1.03 (95% CI 0.12–8.75, *p* = 0.98), showing no effect.

A literature evaluation indicates that chronic renal illness, characterized by creatinine levels exceeding 3.5 mg/dL, correlates with poorer postoperative outcomes [48,57,58]. Creatinine gave OR ≈ 1.66 (95% CI 0.49–5.64, *p* = 0.42) per unit, but no association was found in our study.

Coagulopathy (elevated INR or other coagulation abnormalities) has been identified as a systemic risk factor for anastomotic failure [59,60,61]. The INR had OR ≈ 4.89 (95% CI 0.42–57.4, *p* = 0.21) per unit increase, with very wide confidence limits.

Preoperative serum albumin level—a marker of nutritional status—did not predict leaks in our cohort [OR ≈ 2.41 (95% CI 0.25–23.1, *p* = 0.45)]. Initially, this appears to contradict a significant portion of the surgical literature, which frequently identifies hypoalbuminemia as a risk factor for anastomotic dehiscence and inadequate healing [47,48,62,63,64,65,66,67].

Our study measured albumin only preoperatively, and, importantly, the patient population in both Romanian centers was thoroughly optimized before elective surgery by optimizing nutrition; thus, our institutions may have neutralized hypoalbuminemia as a risk factor. Notably, compared to international studies that mostly describe a link between postoperative hypoalbuminemia and AL, our negative result regarding albumin can be explained by when and in whom the albumin was measured.

Our experience is supported by at least one recent study: Shimura et al. indicated that preoperative albumin levels were not significantly different between patients who had leaks and those who did not; however, a decrease in albumin during the initial postoperative days was strongly correlated with AL [65].

No blood measure demonstrated a significant predictive effect on leak risk, perhaps due to the limited number of events. The impact direction for WBCs and albumin aligns with established risk patterns in the literature [55,64].

These findings suggest that in our cohort of elective colorectal cases, no single preoperative blood parameter was a significant univariate predictor of anastomotic leak. In clinical interpretation, inflammatory markers (WBCs, CRP) and nutritional indicators (albumin, total protein) are significant: elevated WBC counts and diminished albumin have been associated with leaks in bigger studies, but this specific dataset lacked sufficient power to validate this association [55,64].

Clinically, these results reinforce that risk stratification for leaks must integrate multiple factors (patient health, operative factors, etc.) rather than rely on any single lab value. The wide confidence intervals highlight the need for larger studies to determine whether subtle effects of these blood markers truly exist. Both institutions are clinical hospitals equipped to provide comprehensive preoperative optimization, thereby enhancing patients’ physiological status prior to surgery. This capacity for preoperative preparation may influence postoperative outcomes and distinguishes these centers from emergency care hospitals, where such optimization is often limited.

In this retrospective cohort from two Romanian hospitals, a higher ASA (American Society of Anesthesiologists) score and emergency surgical presentation emerged as the most significant predictors of colorectal AL. These findings are strongly corroborated by recent international studies. A 2023 meta-analysis of more than 115,000 colon cancer cases revealed that patients with ASA class ≥III had considerably elevated odds of anastomotic leakage (about 1.3-fold higher), and that urgent (non-elective) surgery similarly heightened the probability of leakage by approximately 30% [14]. The ASA classification adequately represents a patient’s comorbidities and physiological reserve, making its correlation with leak risk understandable; patients who are more ill and weaker endure surgical stress and recover from wounds less efficiently [13,14,23,68,69,70,71,72,73].

Emergency colorectal resections are significantly associated with an increased risk of anastomotic leakage compared to elective procedures [73,74,75]. A study by the American College of Surgeons NSQIP involving about 150,000 colectomy patients (2013–2017) revealed that emergency and urgent cases had greater leak rates compared to elective cases. After additional variables were controlled for, urgent colectomies exhibited almost 30% increased probabilities of anastomotic leakage (adjusted OR ≈1.3, 95% CI 1.2–1.4), whereas emergency colectomies showed about 20% heightened odds (OR ≈1.2, 95% CI 1.1–1.3) in comparison to elective procedures [76]. This outcome is corroborated by our data, indicating that emergency surgery is a strong independent predictor of leakage. The urgent or emergency surgical context, frequently associated with blockage, perforation, or peritonitis, exacerbates risk by limiting preoperative optimization for surgeons and introducing complications such as contamination and hemodynamic instability [46,70,77].

In alignment with our findings, Choi et al. indicated that the conjunction of emergency surgery and elevated ASA class (III–V) poses significant risks, recommending protective strategies such as a diverting stoma or the avoidance of primary anastomosis for patients exhibiting these risk factors [77].

From a clinical perspective, our findings reinforce the need for risk-adapted intraoperative decision-making in high-risk patients. In individuals with ASA class ≥ III or undergoing emergency colorectal resection, the threshold for employing protective strategies—such as diverting stoma creation, staged procedures without immediate anastomosis, or intensified postoperative monitoring—should be lower. This approach is consistent with contemporary evidence identifying high ASA class and emergency surgery as independent predictors of anastomotic leakage and worse postoperative outcomes in colorectal surgery [13,78,79].

In emergency settings, where preoperative optimization and bowel preparation are frequently impossible, careful intraoperative assessments of hemodynamic stability, degree of contamination, and adequacy of tissue perfusion are essential in deciding whether a primary anastomosis is acceptable or whether a damage-control strategy (e.g., Hartmann’s procedure or diversion) is safer [76,80,81].

In our multivariate analysis, neither neoadjuvant radiotherapy (nRT) nor neoadjuvant chemotherapy (nCT) was a significant independent predictor of colorectal AL. To provide context, we analyzed the recent literature investigating the influence of neoadjuvant therapy on the risk of AL. In summary, recent evidence suggests that neoadjuvant radiation is not a conclusive independent predictor of anastomotic leakage following rectal surgery [48,82,83]. The impact of neoadjuvant chemotherapy (excluding radiation) on anastomotic leakage has been examined, particularly in cases with locally advanced colon or rectal cancer treated with comprehensive neoadjuvant therapy. Numerous studies indicate that neoadjuvant chemotherapy does not substantially increase AL rates [47,57,58,84].

This does not contradict the broader literature on nutrition and healing but rather highlights the value of preoperative optimization: it turns a once-risky patient (e.g., with low albumin) into a better prepared surgical candidate.

## 5. Conclusions

In this five-year multicentric retrospective analysis of anterior colorectal resections with primary colorectal anastomosis, emergency surgery and elevated ASA class emerged as the strongest predictors of anastomotic leakage. Preoperative laboratory parameters, including albumin, hemoglobin, inflammatory markers, and renal function indices, were not independently associated with leak occurrence. These findings emphasize the predominant role of baseline physiological status and operative urgency in determining anastomotic integrity.

These insights support a proactive, tailored approach to colorectal surgical planning and highlight the continuing importance of optimizing patients before they ever enter the operating room, thereby improving the chances of uneventful anastomotic healing.

Finally, we acknowledge the limitations of our analysis. This study was retrospective and observational, which introduced inherent biases in data capture and could not prove causation. The sample size, spanning five years but limited to two institutions, was modest. This may have reduced our power to detect subtler risk factors and could explain why some known contributors (e.g., smoking or BMI) did not reach statistical significance in our model.

Additionally, our cohort’s composition—predominantly elective cases—may limit generalizability. Not all healthcare settings will have the resources or time to optimize patients preoperatively, and outcomes can differ in emergency-heavy or resource-limited environments. Moreover, although tumor height in rectal cancer is a recognized technical determinant of anastomotic leakage risk, consistent documentation of the distance from the anal verge was not available in this retrospective dataset, precluding stratified analysis by tumor location. Future prospective studies should systematically record this parameter to enable more precise risk modeling.

Future research, ideally involving large prospective cohorts or pooled international data, is needed to confirm these risk factor trends and to explore interventions for high-risk groups.

## Figures and Tables

**Table 1:** Baseline clinical and treatment characteristics of patients by surgical center.

Characteristic	Colentina (n = 104)	Dr. I. Cantacuzino (n = 49)	*p*-Value
Age, years (mean ± SD)	67.4 ± 9.0	64.0 ± 8.2	0.021
Female sex, n (%)	42 (40.4%)	22 (44.9%)	0.72
Diabetes (yes), n (%)	23 (22.1%)	9 (18.4%)	0.75
Cardiovascular comorbidity (yes), n (%)	63 (60.6%)	32 (65.3%)	0.70
Other comorbidity (yes), n (%)	61 (58.7%)	28 (57.1%)	1.00
Neoadjuvant chemotherapy (yes), n (%)	16 (15.4%)	16 (32.7%)	0.025
Neoadjuvant radiotherapy (yes), n (%)	16 (15.4%)	20 (40.8%)	0.001
Laparoscopic surgery, n (%)	40 (38.5%)	4 (6.1%)	<0.001
Emergency surgery, n (%)	5 (4.8%)	0 (0%)	0.28
Protective stoma (yes), n (%)	3 (2.9%)	1 (2.0%)	1.00
Anastomotic leak, n (%)	11 (10.6%)	5 (10.2%)	1.00

**Table 2:** Univariate analysis of preoperative laboratory markers as predictors of anastomotic leakage.

Variable	Odds Ratio (OR)	95% CI	*p*-Value
Hemoglobin (g/dL)	1.58	0.41–6.12	0.51
WBC (10^3^/μL)	1.68	0.43–6.54	0.45
CRP (mg/L)	1.17	0.14–10.0	0.89
Albumin (g/dL)	2.41	0.25–23.1	0.45
Total Protein (g/dL)	1.03	0.12–8.75	0.98
Creatinine (mg/dL)	1.66	0.49–5.64	0.42
INR	4.89	0.42–57.4	0.21

**Table 3:** Multivariate logistic regression for AL.

Variable	Odds Ratio (95% CI)	*p*-Value
Emergency surgery (vs. elective)	32.2 (4.34–238.0)	0.0007
Neoadjuvant radiotherapy (yes)	4.80 (0.57–40.55)	0.15
Neoadjuvant chemotherapy (yes)	1.09 (0.13–9.24)	0.93

## Data Availability

As per the ethics commission guidelines the original data remain the property of the patients and are only stored in the healthcare system.

## Abbreviations

The following abbreviations are used in this manuscript:

AL	Anastomotic leakage
DM	Diabetes Mellitus
CV	Cardiovascular
ASA	American Society of Anesthesiologists (physical status classification)
BMI	Body mass index
INR	International normalized ratio
KDE	Kernel density estimation
nCRT	Neoadjuvant chemoradiotherapy
OR	Odds ratio
SD	Standard deviation
WBC	White blood cell
CRP	C-reactive protein
